# Supplementary material for: Estimation of regional meteorological aridity and drought characteristics in Baluchistan province, Pakistan
Source: PLoS One. 2023 Nov 30;18(11):e0293073. doi: 10.1371/journal.pone.0293073 (PMC10688865; doi:10.1371/journal.pone.0293073)
Supplement: Attachment — Submitted filename: Review_Report_PONE-D-23-15469.pdf [file pone.0293073.s001.pdf]

Review Report

The manuscript titled "Estimation of Regional Meteorological Aridity and Drought Characteristics of Baluchistan Province, Pakistan" is evaluated and the comments are mentioned below:

The work in the manuscript titled "Estimation of Regional Meteorological Aridity and Drought Characteristics of Baluchistan Province, Pakistan" used the already well-established methods for estimating characteristics of drought and aridity. The introduction section of the manuscript is not written well and there are a lot of confusing statements which make it very difficult to understand. Some of the issues are mentioned under the Specific Comments at the end of this document. The overall text of the manuscript is very confusing and not clear for understanding. I think the authors wrote the manuscript in a rush. The Discussion section of the manuscript discussed the results and therefore it is not look like a scientific discussion. I will suggest the authors to take their time and improve the overall manuscript.

Specific Comments:

1. Page 1, L 24-25: This statement "The temperature and precipitation data were obtained from the Pakistan Meteorological Department" make no sense abstract.
2. Page 1, L 29-30: This statement "The linear regression was used to find out the increasing aridity is increasing in the region" is confusing. Rephrase this for easy understanding.
3. Page 1, L 30-31: The statement "The result revealed that most stations are arid to semi-arid, and the highest increasing aridity is noted in Kalat (0.0065/annum)" can be like "The result revealed that most ***of the*** stations are arid to semi-arid, and the highest increasing aridity is noted in Kalat (0.0065/annum)". This is one of the example of grammatical issues. Please check your manuscript thoroughly for such type of issues. I suggest to edit your manuscript from native English speaker before publication.
4. Keywords: A keyword "Aridity index AI" is mentioned in the list of keywords. It is not clear. Please mentioned clear keyword instead of confused keywords.
5. Page 2, L 56-58: The statements "The severe effects of drought are the negative effects on society and agriculture [8]. According Ofipcc (2013) defined drought as the time of unusually dry weather long enough to create a severe hydrological imbalance" are not clear. In the first statement you are talking about negative effects. What are these negative effects. The second statement is confusing in the start and not understandable.
6. Page 3, L 71-72: The statement "The Asian counties …". I think you mean countries. Check your manuscript thoroughly for such type of issues.
7. Page 3, L 76-78: The statement "Less than 250 mm of rain falls annually throughout the entire province of Sindh, the majority of Balochistan, large portions of the Punjab, and

the central regions of the Northern Areas [16]” is not clear. Rephrase it and make it clearer for easy understanding.

8. Page 8, L 178: Eq. (6) is not clear. Please check all equations of your manuscript thoroughly.
