# Supplementary material for: Estimation of regional meteorological aridity and drought characteristics in Baluchistan province, Pakistan
Source: PLoS One. 2023 Nov 30;18(11):e0293073. doi: 10.1371/journal.pone.0293073 (PMC10688865; doi:10.1371/journal.pone.0293073)
Supplement: Attachment — Submitted filename: Response letter PLoS ONE 9-7-23.docx [file pone.0293073.s002.docx]

**Ref.**  **ESPR-D-21-18794R1: Estimation of regional meteorological aridity and drought characteristics of Baluchistan province, Pakistan**

**Point to Point Response to the Reviewer Comments**

We are thankful to the editor and team for their timely feedback. We are also thankful to reviewer for their deep and valuable comments that improved the quality of the manuscript. We are submitting the point-by-point response to the comments and an updated manuscript file with highlighted **revision as suggested by worthy reviewers**. We are hopeful that the revised manuscript has improved to the level of satisfaction and approval. We also hope that now the manuscript meets the journal’s publication requirements.

**Editor**

1. Thank you for stating the following financial disclosure:

This study is supported by National Natural Science Foundation of China (Grant No. 41877433) and the Hebei Natural Science Foundation and Key Basic Research (Grant No. 18963301D).

At this time, please address the following queries:

**Author Response: The funders had no role in study design, data collection and analysis, decision to publish, or preparation of the manuscript.**

2. We note that Figures 1,2, 5 and 6 in your submission contain [map/satellite] images which may be copyrighted. All PLOS content is published under the Creative Commons Attribution License (CC BY 4.0), which means that the manuscript, images, and Supporting Information files will be freely available online, and any third party is permitted to access, download, copy, distribute, and use these materials in any way, even commercially, with proper attribution. For these reasons, we cannot publish previously copyrighted maps or satellite images created using proprietary data, such as Google software (Google Maps, Street View, and Earth). For more information, see our copyright guidelines: <http://journals.plos.org/plosone/s/licenses-and-copyright>.
   We require you to either (1) present written permission from the copyright holder to publish these figures specifically under the CC BY 4.0 license, or (2) remove the figures from your submission:

**Author Response: The figures 1, 2, 5 and 6 in our submissions are the author own work and further a copy right holder letter uploaded to fulfil the journal requirements. The figures are modified after Rafiq M, Li YC, Cheng Y, Rahman G, Ali A, et al. (2022) Spatio-statistical analysis of temperature and trend detection in Baluchistan, Pakistan. Ecological Questions 33: 67-78. The research paper is published in under a Creative Commons Attribution License and the authors have right to publish or reprint it.**

3. Please note that PLOS ONE has specific guidelines on code sharing for submissions in which author-generated code underpins the findings in the manuscript. In these cases, all author-generated code must be made available without restrictions upon publication of the work. Please review our guidelines at <https://journals.plos.org/plosone/s/materials-and-software-sharing#loc-sharing-code> and ensure that your code is shared in a way that follows best practice and facilitates reproducibility and reuse.

**Author Response: No coding used in this manuscript.**

4. We note that the grant information you provided in the ‘Funding Information’ and ‘Financial Disclosure’ sections do not match. Please see below for your reference.

**Author Response: This study is supported by National Natural Science Foundation of China (Grant No. 41877433) and the Hebei Natural Science Foundation and Key Basic Research (Grant No. 18963301D).**

**Also added in the Cover Letter**

5. Please complete your Competing Interests on the online submission form to state any Competing Interests. If you have no competing interests, please state "The authors have declared that no competing interests exist.", as detailed online in our guide for authors at <http://journals.plos.org/plosone/s/submit-now>
    This information should be included in your cover letter; we will change the online submission form on your behalf.

**Author Response: Updated and also added in the cover Letter as “The authors have declared that no competing interests exist.”**

6. We note that Figures 1,2, 5 and 6 in your submission contain [map/satellite] images which may be copyrighted. All PLOS content is published under the Creative Commons Attribution License (CC BY 4.0), which means that the manuscript, images, and Supporting Information files will be freely available online, and any third party is permitted to access, download, copy, distribute, and use these materials in any way, even commercially, with proper attribution. For these reasons, we cannot publish previously copyrighted maps or satellite images created using proprietary data, such as Google software (Google Maps, Street View, and Earth). For more information, see our copyright guidelines: <http://journals.plos.org/plosone/s/licenses-and-copyright>.

**Author Response: The Content Permission form uploaded. These figures are prepared by one of the author and there is no restriction on its distribution and reprinting or publishing.**

7. Please ensure that you refer to Table 2 and Table 5 in your text as, if accepted, production will need this reference to link the reader to the Table.

**Author Response: Reference to Table 2 and Table 5 added in the revised manuscript.**

**Reviewer I**

1. The paper is well organized and well written according to the journal format. English language is good however, I have following concerns before accepting this manuscript.

**Author Response: Thank you for your positive feedback. We revised the manuscript carefully.**

2. Dear authors, I found some grammatical and technical issues in the abstract section therefore I will suggest to rewrite and align it with international journal standards.

**Author Response: We revised the abstract and tried to remove all the grammatical and technical issues. Most of the portion is rewritten to meet the international journal standard.**

3. The given keywords are only three in numbers and there should be at least five keywords.

**Author Response: More keywords are added in the revised manuscript.**

4. I will suggest to improve the writing of the introduction section and also include some further latest and updated literature regarding drought and aridity in the region.

**Author Response: The introduction section has been improved with more latest and updated literature.**

5. In the study area section “From a morphological perspective, the study area (Baluchistan province) is a mountainous, arid in southwest territory of Pakistan (Fig .1).” morphology is not an accurate word. Replace it with some more suitable word.

**Author Response: The study area section has been revised and the inappropriate words have been removed in the revised section.**

6. Correct the given latitude and longitude in the study area section “The region is located between 30.12 oN and 67.01 oE latitude and longitude, respectively.”

**Author Response: The latitude and longitude in the study area section has been revised.**

7. There is a lot of grammatical and technical mistakes in the study area section like (i) “and are covered in dry terrain, are west of the Baluchistan plains.” (ii) “The bulk of the province is covered by the Kharan desert in the southwest” (iii) “Additionally, the province is crucial geographically for both regional trade and security.” (iv) “Baluchistan makes up 4% of Pakistan's overall land mass” (v) “Due to terrain brought on by monsoon and western storms, there are variations in rainfall and temperature dispersion.” (vi) “Baluchistan primarily experiences erratic weather that ranges from 30 mm to 397 mm annually”.

**Author Response: Thank you worthy reviewer for pointing out these errors and mistakes in our manuscript. We carefully revised the manuscript and removed all these issues. We hope that the revised manuscript will be according to the language structure and all technical errors have been removed.**

8. Figure 1 needs to be corrected as it does not reflect the actual topography of the study area.

**Author Response: Figure 1 has been revised**

9. Rewrite the first sentence of the data collection and method section.

**Author Response: We revised this section**

10. Rewrite the caption of table 1. Rewrite the caption of table 2. Rewrite table 3 caption. Rewrite the caption of table 4. Rewrite the caption of table 5. Rewrite the caption of Figure 4. Rewrite the caption of Figure 5.

**Author Response: We revised all captions of the tables and figures**

11. If this manuscript is based on SPEI then why the authors are focusing on SPI in the methodology section.

**Author Response: We revised this section of the manuscript**

12. The discussion section needs to be carefully studied and revised in light of more scientific literature with analytical reasoning.

**Author Response: We carefully rewrite the discussion section and relate the results with latest and updated literature with more analytical reasoning.**

13. The conclusion section needs proofreading for grammatical correction

**Author Response: We revised the Conclusion section very carefully to remove the grammatical errors.**

**Reviewer II**

1. The work in the manuscript titled “Estimation of Regional Meteorological Aridity and Drought Characteristics of Baluchistan Province, Pakistan” used the already well-established methods for estimating characteristics of drought and aridity. The introduction section of the manuscript is not written well and there are a lot of confusing statements which make it very difficult to understand. Some of the issues are mentioned under the Specific Comments at the end of this document. The overall text of the manuscript is very confusing and not clear for understanding. I think the authors wrote the manuscript in a rush. The Discussion section of the manuscript discussed the results and therefore it is not look like a scientific discussion. I will suggest the authors to take their time and improve the overall manuscript.

**Author Response: Thank you respected reviewer for your encouraging words. We revised the whole manuscript in light of the worthy reviewer comments and hopefully now it will be in much improved form.**

2. Page 1, L 24-25: This statement “The temperature and precipitation data were obtained from the Pakistan Meteorological Department” make no sense abstract.

**Author Response: Removed this sentence from the revised manuscript.**

3. Page 1, L 29-30: This statement “The linear regression was used to find out the increasing aridity is increasing in the region” is confusing. Rephrase this for easy understanding.

**Author Response: We rephrased all such confusing sentences in the revised manuscript. Hopefully you will find it in much better form.**

4. Page 1, L 30-31: The statement “The result revealed that most stations are arid to semi-arid, and the highest increasing aridity is noted in Kalat (0.0065/annum)” can be like “The result revealed that most of the stations are arid to semi-arid, and the highest increasing aridity is noted in Kalat (0.0065/annum)”. This is one of the example of grammatical issues. Please check your manuscript thoroughly for such type of issues. I suggest to edit your manuscript from native English speaker before publication.

**Author Response: It has been revised to clarify the results and statement in the manuscript and avoid any confusing statements.**

5. Keywords: A keyword “Aridity index AI” is mentioned in the list of keywords. It is not clear. Please mentioned clear keyword instead of confused keywords.

**Author Response: Keywords have been revised. Hopefully it will be according to your suggestions.**

6. Page 2, L 56-58: The statements “The severe effects of drought are the negative effects on society and agriculture [8]. According Ofipcc (2013) defined drought as the time of unusually dry weather long enough to create a severe hydrological imbalance” are not clear. In the first statement you are talking about negative effects. What are these negative effects. The second statement is confusing in the start and not understandable.

**Author Response: We revised all these sentences and added more scientific literature. Removed all the grammatical and technical errors from the manuscript.**

7. Page 3, L 71-72: The statement “The Asian counties …”. I think you mean countries. Check your manuscript thoroughly for such type of issues.

**Author Response: Yes, respected reviewer the word is countries and corrected in the revised manuscript.**

8. Page 3, L 76-78: The statement “Less than 250 mm of rain falls annually throughout the entire province of Sindh, the majority of Balochistan, large portions of the Punjab, and the central regions of the Northern Areas [16]” is not clear. Rephrase it and make it clearer for easy understanding.

**Author Response: We revised this section of the manuscript. “Most of the Baluchistan, Sindh and the southern and central parts of Punjab receives less than 250 mm of rainfall annually”.**

9. Page 8, L 178: Eq. (6) is not clear. Please check all equations of your manuscript thoroughly.

**Author Response: It is checked and revised**
