# Supplementary material for: Estimation of regional meteorological aridity and drought characteristics in Baluchistan province, Pakistan
Source: PLoS One. 2023 Nov 30;18(11):e0293073. doi: 10.1371/journal.pone.0293073 (PMC10688865; doi:10.1371/journal.pone.0293073)
Supplement: Attachment — Submitted filename: Response letter PLoS ONE 30-9-23.docx [file pone.0293073.s003.docx]

**Ref.**  **ESPR-D-21-18794R1: Estimation of regional meteorological aridity and drought characteristics of Baluchistan province, Pakistan**

**Point to Point Response to the Reviewer Comments**

We are thankful to the editor and team for their timely feedback. We are also thankful to reviewer for their deep and valuable comments that improved the quality of the manuscript. We are submitting the point-by-point response to the comments and an updated manuscript file with highlighted **revision as suggested by worthy reviewers**. We are hopeful that the revised manuscript has improved to the level of satisfaction and approval. We also hope that now the manuscript meets the journal’s publication requirements.

**Editor**

1. Thank you for stating the following financial disclosure:

This study is supported by National Natural Science Foundation of China (Grant No. 41877433) and the Hebei Natural Science Foundation and Key Basic Research (Grant No. 18963301D).

**Reviewer I**

1. Reviewer 1. The authors have addressed all my comments. Therefore, I accept this manuscript to be published in PLOS One Journal.

**Author Response: Thank you for your positive feedback and accepting our manuscript for publication in this prestigious journal.**

**Reviewer 3**

This paper reports the Estimation of Regional Meteorological Aridity and Drought Characteristics of Baluchistan Province, Pakistan. The aim of this study was to examine extreme climatic conditions such as drought in the province of Baluchistan. Overall, the themes are interesting as they show the climatic situation in this province in order to prepare the government to take adaptation and mitigation decisions to counter the effects of climate change on local populations. I suggest Major revision.

**Author Response: Thank you respected reviewer for your encouraging words. We revised the whole manuscript in light of the worthy reviewer comments and hopefully now it will be in much improved form. Indeed it will be helpful in devising policies to combat drought in this arid zone of Pakistan.**

1. noticed that there is no regionalization analysis in this study, despite the reference in the title: "Estimation of regional meteorological aridity and drought characteristics in Baluchistan province, Pakistan". Therefore, a regionalization study using multivariate statistics such as PCA, Ward clustering with Ecludian distance, k-means or Moran's spatial autocorrelation...etc., should be added.

**Author Response: We tried our best in the revision of this manuscript. We added further analysis as you suggested but unfortunately the number of meteorological stations are scarce in this region so this type of regionalization was not possible.**

2. Numerous studies on drought in Pakistan have been reported in the literature, but the authors do not sufficiently summarize them in the introduction, nor do they show how the study carried out is new compared to those that preceded it, which are, for example, the following:
   - Ahmed, K., Shahid, S., &Nawaz, N. (2018). Impacts of climate variability and change on seasonal droughtcharacteristics of Pakistan. Atmospheric Research. doi:10.1016/j.atmosres.2018.08.020
   - Jamro, Shoaib, FalakNazChanna, Ghulam Hussain Dars, Kamran Ansari, and Nir Y. Krakauer. 2020. "Exploring the Evolution of DroughtCharacteristics in Balochistan, Pakistan" Applied Sciences 10, no. 3: 913. <https://doi.org/10.3390/app10030913>
   -Jamro, Shoaib, Ghulam Hussain Dars, Kamran Ansari, and Nir Y. Krakauer. 2019. "Spatio-Temporal Variability of Drought in Pakistan Using Standardized Precipitation Evapotranspiration Index" Applied Sciences 9, no. 21: 4588. <https://doi.org/10.3390/app9214588>
   -Dilawar, Adil, Baozhang Chen, Arfan Arshad, Lifeng Guo, Muhammad Irfan Ehsan, Yawar Hussain, Alphonse Kayiranga, Simon Measho, Huifang Zhang, Fei Wang, and et al. 2021. "TowardsUnderstanding Variability in Droughts in Response to Extreme Climate Conditions over the Different Agro-Ecological Zones of Pakistan" Sustainability 13, no. 12: 6910. <https://doi.org/10.3390/su13126910>

**Author Response: We added the suggested literature and summarize them in the Introduction section. We also highlighted how this study is different from the already conducted research studies in this region.**

3. In this manuscript, neither drought characteristics such as drought duration or intensity, nor the maximum interannual variation of the SPEI or the aridity index have been carried out according to the meteorological stations of the present contribution.

**Author Response: It has been revised to and drought characteristics like drought duration, intensity and severity results have been added in the revised manuscript.**

4. As with other drought indices, a long reference period (30-50+ years) is required to increase the reliability of the SPEI drought estimate. However, the Barkhan station does not meet this condition (2009-2021). Drought analyses for this station should be taken with great caution or excluded from the study.

**Author Response: The results of Barkhan has been removed from the manuscript as suggested by worthy reviewer.**

5. All the results of your analyses are not compared with those found in the literature.

**Author Response: We revised all discussion section and tried to incorporate further discussion in light of previous literature.**

6. Why haven't you carried out a quality control study on your data, for example to homogenize precipitation data, identify temperature extremes, etc.? It would be necessary to add descriptive statistics to your data before calculating the SPI and the aridity index.

**Author Response: Descriptive statistics have been added in the revised manuscript.**

7. In the discussion section, the authors do not show how the aridity index differs from the SPEI index in your analyses.

**Author Response: We tried to differentiate the Aridity Index and SPEI in the revised manuscript.**

8. Finally, the SPEI can be calculated on a time scale ranging from 1 to 48 months. I'd like to know why you prefer SPEI at 1 and 6 months.

**Author Response: in this manuscript we mainly focused on short term drought events therefore we used 1 and 6-months SPEI in this manuscript.**

**Minor but important issue:**

1. In the summary, line 29-30, please rewrite the sentence.

**Author Response: We revised the suggested sentences.**

2. In the summary, line 31, replace (0.0065/year) with year to clarify things for readers.

**Author Response: We revised it as suggested by the worthy reviewer.**

3. In the abstract, SPEI at 1 and 6 months, rewrite as SPEI at 1 and 6 months.

**Author Response: Rewritten as suggested by the worthy reviewer.**

4. In the abstract, remove the hyphen before the expression aridity (Key words: - Aridity).

**Author Response: Hyphen removed from the Keywords.**

5. In the introduction, delete the second comma after line 59. The same applies to line 65.

**Author Response: Comma Deleted**

6. Please quote a reference for the remark in line 71 of the introduction.

**Author Response: Reference added to the mentioned sentence.**

7. In the figure on line 111, please replace the unit of elevation with the meter, which is more fluid and easier to understand. Then, a more gradated coloring will be useful to highlight the differences in terrain leveling according to the geographical positions of the weather stations.

**Author Response: The figures are revised as per your valuable suggestions.**

8. Please rewrite lines 115 to 117, as the text explanation is not fluent, and specify the type of data: daily or monthly.

**Author Response: We revised it as suggested by the worthy reviewer.**

8. Line 119: (Table.1), rewrite as (Table 1).

**Author Response: Corrected as per given suggestions.**

10 . Please specify the software used and its reference in your study.

**Author Response: Software added and discussed.**

11. On line 129, replace "the dot" with a "colon" after the reference and number the equation.

**Author Response: dot removed and colon added after reference and number has been added to the equation.**

11 . On lines 145-147, a reference is required.

**Author Response: We added reference as suggested by the worthy reviewer.**

12 Lines 149 and 151, a reference is required.

**Author Response: We added reference as suggested by the worthy reviewer.**

13 Line 152, the equation is not the first but the second for numbering, please rectify for all others.

**Author Response: corrected.**

14 The equation on line 178, please review the question marks in the formula.

**Author Response: corrected.**

15 For each equation, a reference is required.

**Author Response: Reference is given.**

16 10. In figure 3, line 279, please add the respective p. values to the trend curves for the aridity and SPEI indices.

**Author Response: P values are added in trend curve. Thank you once again for your time and valuable suggestion.**
